# “It's a good distraction from the mayhem of reality”: a reflexive thematic analysis on the role of video games to support coping during a crisis

**DOI:** 10.3389/fdgth.2025.1608322

**Published:** 2025-08-18

**Authors:** George Farmer, Nina Higson-Sweeney, Chris Fullwood

**Affiliations:** ^1^ Department of Psychology, School of Social Sciences, University of Westminster, London, United Kingdom; ^2^ Department of Psychology, University of Bath, Bath, United Kingdom; ^3^ Department of Psychology, Birmingham City University, Birmingham, United Kingdom

**Keywords:** video gaming, coping, psychological well-being, stress, crisis

## Abstract

**Introduction:**

Video games have been shown to offer psychological benefits to gamers during times of stress. One recent, salient example of a stress-inducing event was the COVID-19 pandemic, which created periods of social isolation and uncertainty on a global scale. The current study offers a glimpse into the lives of video gamers during the COVID-19 pandemic and the role of video games in navigating day-to-day stresses during a crisis.

**Methods:**

Participants (*n* = 13) were recruited online via volunteer sampling and conducted text-based structured interviews via Skype or via email. Using an interview guide developed for this study, participants were asked questions regarding demographic traits such as age, gender, and employment status, as well as questions regarding their current video game play at the time of the interview and general questions, such as whether video games offered any perceived benefits during the COVID-19 lockdown.

**Results:**

Reflexive thematic analysis of 13 interviews generated three themes that focus on video games, coping, and well-being. For many participants, video games provided what the lockdown took away: permitting the restoration of agency, community, and a sense of purpose. Immersing oneself in video games was a proactive coping mechanism for some but protective for others, suggesting a range of strategies that likely differed in effectiveness. Finally, gamers discussed the tension between viewing gaming as a beneficial and valuable activity versus unproductive time-wasting.

**Discussion:**

Qualitative results are evaluated through the lens of self-determination theory. The use of video games to deal with the day-to-day stresses of the COVID-19 pandemic illustrates teachable moments that speak to video gaming as a compensatory activity during times of crisis, which applies to future global health events beyond COVID-19.

## 1 Introduction

The psychological ramifications of the COVID-19 pandemic continue to be felt many years after social distancing measures were lifted. Several phases of social lockdowns were implemented within the UK, starting in March 2020, before being fully rescinded in July 2021 (1). Early commentators raised concerns over the potential long-term negative physical and psychological impacts that social lockdowns might have (2–5). Indeed, these concerns were realised during April 202, with a 10.8% reduction in reported general psychological well-being scores in the UK compared to scores measured between January 2017 and May 2019. Moreover, this reported reduction was 8.1% worse than initially predicted (6). Research suggests that the lockdowns presented individuals with numerous stress-inducing risk factors, such as needing to adapt to new ways of working and increased feelings of job insecurity (6, 7), concerns over food/water/shelter scarcity (8), and general financial insecurities (7), all of which contributed to a perceived *mental health crisis* (3). While the COVID-19 pandemic was certainly a modern-day crisis, on reflection, we argue that this is a singular use case that has teachable lessons for future social crises or other moments of widespread social upheaval.

People naturally orient themselves towards creating and maintaining social relationships, which offer emotional support and physical protection (9). The emotional distress of relationship breakdowns is particularly salient (10). Stressors associated with experiencing the loss of social connections are among some of the strongest predictors of emotional distress (11) and may increase the sensitivity to subsequent stressors involving loss in the future (12). This is reflected in research conducted towards the beginning of the lockdown, with younger individuals reporting worse scores across several mental health questionnaires compared to older individuals, possibly due to a significant decline in freedom of movement and consequent strain on maintaining social connections that would contribute to a negative impact on well-being (2, 13, 14).

When individuals feel overwhelmed with stress, there is a tendency to gravitate towards activities that facilitate *stress-coping* (14). According to Lazarus and Folkman's Transactional Model of Stress and Coping (15), stress results from an individual interacting with their environment in a way that exceeds their physical or psychological resources and endangers their well-being (16). This comprises two phases: (1) cognitive appraisal and (2) coping. The dual-phasic process involves cognitive assessment of the threat (i.e., the stressor) and appraisal of whether the individual can use the resources at their disposal to cope with the stressor, which in turn relies on an individual ability to mitigate, reduce, or tolerate the physical or psychological demands created by the stressor (16). This framework is particularly relevant to those who experienced distress due to the lockdown, as individuals were facing a situation they had not experienced before and had little agency over controlling their circumstances, which would exceed most individuals' resources and demonstrably jeopardise their well-being.

Looking at specific factors contributing to stress relief, previous work has suggested that performing tasks that provide a sense of individual mastery is associated with better health outcomes (17). This has been supported by reviews of coping literature (18) and included as a part of positive coping frameworks (19). As well as mastery, positive self-esteem, social support, and optimistic expectations are all coping resources associated with improved psychological health (18). There are a range of media-based activities associated with stress-coping, including watching television (20), using the Internet (21), and playing video games (22). Therefore, it is not unreasonable to suggest that activities like video gaming may have helped to buffer the negative effects of the COVID-19 lockdown.

Many articles within video game psychology suggest negative health and personal consequences of participating in this form of media, with a push to pathologize this behaviour (23). For example, previous studies have proposed links between video gaming and addiction (24, 25), depression (26), and stress (27), as well as a variety of negative personal outcomes such as poorer academic performance (28). The most recent example of this is the inclusion of Internet Gaming Disorder into the ICD-11 by the World Health Organization [WHO; (29)]. However, much of this literature derives from self-report, cross-sectional data (23, 30), and most notably, addiction-based research articles have been critiqued for over-reporting false positive cases of video game addiction amongst an ongoing empirical debate regarding the nature of gaming addiction as a construct (23, 30, 31). Considering this, the reliability of these data may be called into question, and further investigation is needed, taking a bottom-up approach.

Against this backdrop, more recent research has explored the positive health benefits of video gaming (22, 32–34). Such work describes the potential for video games, particularly those played online, to encourage social interaction, which is reported to have benefits for psychological well-being (35), reduce psychological symptoms of illnesses such as depression (36), and are reported to be used as a popular form of mood management to recover from psychological stress (37). Video gaming can also be used as a distraction from potentially harmful ruminations about stressful life events (38), assisting in the fulfilment of basic psychological needs such as autonomy (the need for agency over one's own life), competence (the need to experience control over and mastery in a domain), and relatedness (the need to feel connected to and cared for by others (39, 40). This is supported by previous research, which suggests that video gaming has the potential to provide players with a sense of mastery and fulfil basic psychological needs (41–43).

These three basic psychological needs are core principles from a popular theory of human motivation known as self-determination theory [SDT; (39)]. Self-determination theory has been used as a theoretical basis for many studies of video game use (43–48), with many suggesting that the affordances of video games as a medium can fulfil these psychological needs through meaningful, or eudaimonic, entertainment experiences (22, 49–52). There are also unique features of video gaming that can enhance need satisfaction. Tamborini et al. (53), found that engaging in multiplayer or social-oriented video game experiences increased relatedness satisfaction and experiencing improved video game control interfaces increased competence satisfaction (51). This was emphasised within the COVID-19 lockdown, with enforced social isolation placed on several nations worldwide as a preventative measure to inhibit the spread of the coronavirus. Indeed, the pandemic provided opportunities for individuals to increase their frequency of video gaming (54), engage in social interactions that transcended physical barriers (55), and relieve stress (54–57).

Qualitative work in this area suggests that video gaming has a positive effect on the overall well-being of video gamers and is a viable option for stress coping. There have been several qualitative studies that suggest *Animal Crossing* was a popular video game during lockdown, allowing for the fulfilment of basic psychological needs by offering a chance for players to explore their freedom of expression to satisfy autonomy needs, setting goals to enhance their feelings of competence, whilst also offering psychological relief from stress (58–61). While some researchers raised concerns about the potential for addictive behavioural patterns of video game use during the pandemic (62), video gamers were also supported by video game companies to follow health guidelines issued by the WHO.

Indeed, in 2020, several major video game studios such as *Microsoft*, *Ubisoft*, and *Sega* partnered with the WHO to encourage the principles of distanced play and cooperation using the hashtag *#PlayApartTogether* on Twitter (now known as “*X*”) to encourage a public-facing message of preventing the spread of the virus (63, 64). Video gamers were also observed using promotional offers from video game companies to gift video games to their friends or family, convincing traditional non-video gamers to participate as a collective (65), which further encouraged positive well-being by completing enjoyable activities with others.

In the academic field, there is a clear need to understand how video games might help or hinder our ability to cope with stressful life circumstances at an individual level. This opportunity presented itself during the COVID-19 pandemic: to document the lived experiences of video gamers in a powerfully salient stress-inducing environment and explore this phenomenon through a psychological lens. While there have been articles published on the experience of using video games during the lockdown since the end of the social lockdown in the UK [e.g., (66–68)], an opportunity for more qualitative research exists to provide deeper insights into the lived experiences of video game players (60) and how they perceive gaming influences their responses to stressful events (69).

The results of this study will be able to add to the existing literature, providing further examples of how individuals can use video gaming to satisfy basic psychological needs and maintain social connections in times of physical and social isolation, sudden changes to day-to-day living, and pressures brought on by financial and health uncertainties. By examining the lived experiences of video gamers during the height of the COVID-19 pandemic, we expect findings from this investigation to be applicable to understanding the coping behaviours of video gamers during a variety of different types of stress-inducing events.

## 2 Materials & methods

### 2.1 Procedure

Due to the constraints of the lockdown, this qualitative interview-based study was conducted online, with all interviews taking place during June 2020. The research question guiding this study was: *How did individuals use video gaming to navigate the psychological stressors created by the COVID-19 pandemic?* Participants were recruited through advertisements on social media networks and had the option of conducting structured interviews either by email (*n* = 6) or by *Skype* (*n* = 7), an online messaging service, via the chat function. This research was approved by the University of Wolverhampton's research ethics committee.

Participants were asked to confirm that informed consent had been given prior to starting the interview. Using an interview guide developed for this study (see Supplementary Materials), participants were asked questions based on demographic information, such as age, gender identity, employment status, and whether they were considered a “key worker” during the lockdown. Participants were then asked questions relating to their current video game play (e.g., “How many hours do you spend playing video games per week?”, “What would you say are your top three video game titles during quarantine?”) and general video game questions (e.g., “What kinds of benefits would you say video games offer you, compared to other digital mediums such as social media?”).

Interviews conducted on *Skype* lasted approximately 30–60 min, whereas individuals who participated via email were sent the structured interview questions and asked to send their replies to the lead researcher (GF) at a time convenient to them. The interviewing stage of the study concluded when the researchers agreed that enough data had been collected to answer the research question and begin the process of thematic analysis.

### 2.2 Participants

A mix of UK and US participants were recruited using volunteer sampling methods, with individuals who indicated an interest in participating in a qualitative study about their experiences with video gaming during the COVID-19 lockdown contacting the third author prior to arranging a research interview. There were no restrictions on who could participate, other than that they must be over 18 years old and have played video games actively (i.e., more than one day per week) during the lockdown. Interviews were conducted with 13 participants between the ages of 20 and 42 (*M* = 28.38, *SD* = 6.56), with the majority identifying as male (61.5%). An information power approach was used to determine when data collection procedures would stop (70), with a moderate level of information power indicated by the model presented by Malterud et al. Using the example provided by Malterud et al., an initial cautious estimate of 10 participants was targeted (70), with additional data gathered to increase the potential range of experiences discussed by participants. However, it should be noted that data was gathered from an unintentionally specific cluster of individuals (mainly males aged 20–30), which does not capture the full range of experiences during the COVID-19 pandemic and may omit perspectives from individuals of other genders, age groups, and cultural backgrounds.

Participants indicated that they spent between three and thirty hours gaming every week (*M* = 13.77), which could be spread across two to seven days (*M* = 5.08). At the time of the interviews during lockdown, six participants were in current employment, five were students, one was furloughed, and one did not disclose their employment status. To protect participant anonymity, all participants were assigned pseudonyms, which are used throughout the manuscript when quoting or referring to participants. See Table 1 for an overview of demographics by participant.

**Table 1:** Participant demographics and characteristics.

Pseudonym	Age	Employment during lockdown	Hours spent gaming per week	Days spent gaming per week^a^
Adam	30–39	Employed	3–4	Every day
Bella	20–29	Student	18	Most days
Chloe	20–29	Student	6–12	Some days
Emily	20–29	Student	25	Every day
Jackson	20–29	Student	12	Some days
James	30–39	Employed	15	Most days
Kieran	30–39	Self-employed	14	Most days
Mia	20–29	Employed	20–30	Most days
Paige	20–29	Employed	10	Most days
Ryan	20–29	Furloughed	15	Some days
Stephen	30–39	Not disclosed	7	Every day
Will	20–29	Redundancy, then Employed	22	Every day

^a^ Days spent gaming coded as: 1 = one day; 2–4 = some days; 5–6 = most days; 7 = every day.

### 2.3 Data analysis

Data were analysed using reflexive thematic analysis (71, 72), a method for systematically identifying, analysing, and reporting shared patterns of meaning across a qualitative dataset, focusing on reflexivity. The analysis was inductive, meaning that it was driven by the contents of the data and not approached with a pre-existing theoretical framework in mind. An experiential approach underpinned the analysis, focusing on participants' lived experiences and understanding how they make sense of their worlds.

Analysis of the interview transcripts was guided by Braun and Clarke's six-phase process (71) and was led by one author. In phase one, [Second Author] read and re-read the transcripts to become familiar with the data, alongside taking notes of initial points of interest. In phase two, [Second Author] undertook line-by-line coding of the transcripts using NVivo software. Coding was done at a semantic (i.e., participant-driven) and latent level (i.e., researcher-driven). In phase three, [Second Author] began to combine codes to generate initial themes; each focused on addressing the study's main research question. This process involved [Second Author] reading through all codes and associated quotes and identifying potential areas of overlap or similarity around a central organising concept. These codes were then subsumed under tentative theme and subtheme headings, and continually rearranged and reworked until a clear set of initial themes had been generated that adequately addressed the study research question. In phases 4 and 5, [Second Author] consulted with [First Author] and [Third Author] to review and refine the initial themes, which included developing theme titles and summaries of what was included. As reflexive thematic analysis does not incorporate coding reliability, a formal inter-rater reliability process was not followed. Instead, the research team discussed the identified codes, associated quotes and subsequent themes during team meetings, with [First Author] and [Third Author] using their knowledge of the interviews and familiarity with the data to consider whether [Second Author's] interpretations reflected their own understandings of the data. Any disagreements were resolved during these meetings. In phase six, [Second Author] wrote up the results, including representative quotes from across the sample to evidence and illustrate the interpretations. During this phase, [First Author] and [Third Author] provided verbal feedback during team meetings, and written feedback on drafts of the manuscript.

### 2.4 Reflexivity

Reflexivity refers to the process of researchers critically examining how their positionality may have influenced research conducted and is an integral part of reflexive thematic analysis that distinguishes it from alternative approaches that focus on codebooks and reliability (72, 73). All three authors identify as research psychologists, with [First Author] and [Third Author] specialising in cyberpsychology and video games research, and [Second Author] specialising in youth mental health and qualitative methods. All three authors identify as video gamers and saw an increase in their gaming during the COVID-19 pandemic, similar to many of the participants.

[Second Author] kept a reflexive diary throughout the analytic process to consider how their experiences, values and beliefs may have influenced their interpretation of the data and presentation of the findings. This was discussed in team meetings with [First Author] and [Third Author], particularly during phases three, four and five, where team-based analytic discussions helped to inform the final results. During this process, the research team recognised that we had largely positive experiences of using video games as a coping mechanism during the pandemic, which was what initially drove our interest in this topic and our motivation to explore this area. Because of this, we were keenly aware that we might focus on experiences that mirrored our own, and neglect or not recognise incongruent experiences. We kept this in mind as we iteratively moved between the different phases of reflexive thematic analysis, re-reading the coded transcripts to ensure that all relevant data had been captured.

## 3 Results

### 3.1 Themes

**Figure 1:**
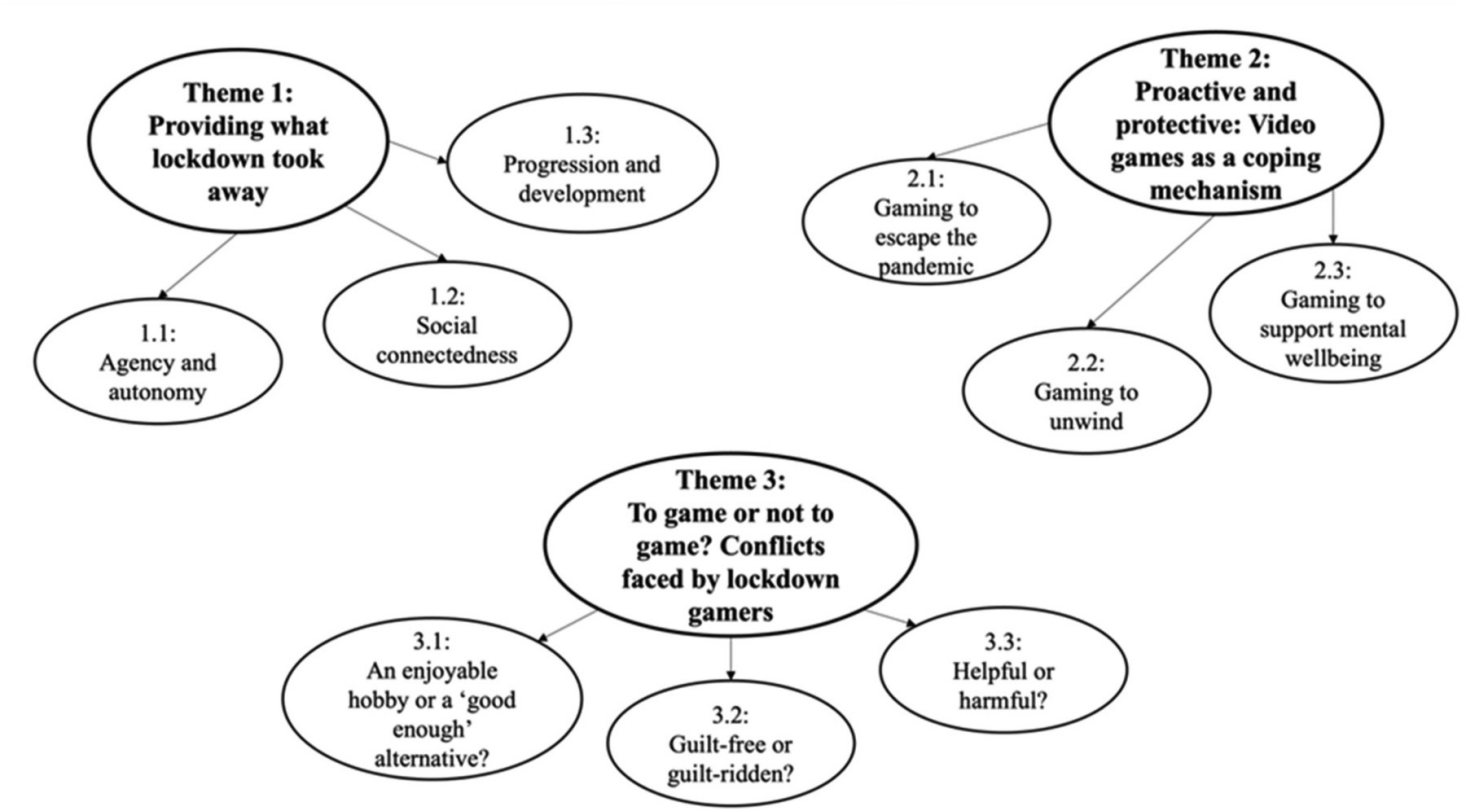
Thematic map.

Analysis of the data generated three themes that collectively showcase video gamers' experiences during the COVID-19 lockdown and the impact of video games on their mental well-being (see Figure 1 for a thematic map).

#### 3.1.1 Theme 1: providing what lockdown took away

Participants described how playing video games during lockdown provided them with the opportunity to engage with experiences that the imposed restrictions had made difficult or impossible. This is explored through three subthemes: (1.1) Agency and autonomy, (1.2) Social connectedness, and (1.3) Progression and development.

##### 3.1.1.1 Agency and autonomy

A core element of many COVID-19 restrictions governments imposed worldwide were limitations on where people could go and what they could do. This limited people's sense of freedom and the level of control they felt they had over their lives (74, 75). In comparison, video games seemed to allow players to regain this lost sense of control through the restoration of agency and autonomy to make decisions in these virtual settings. This is congruent with the fulfilment of autonomy, a basic psychological need according to SDT (76). Participants discussed how this was made possible across various genres, including roleplaying games (RPGs), online multiplayer strategy games, and simulators.

“In ACNH [Animal Crossing New Horizons] you choose what to do with your island and what villagers you have. In League [of Legends] you choose what game mode, and depending on that, what character you play with. In Persona 5, you have the goal and a deadline but when you meet the deadline is up to you as long as it is met. You also get periods of free time where you choose what to do. Having that autonomy is really cool.” (Mia)

While participants acknowledged that many games include some level of restriction, particularly ones that are more linear and plot-driven, they still experienced enough freedom of choice to feel in control. For some, this seemed related to how immersed they felt in the game, with increased personalisation and player-driven narratives heightening this sensation.

“I am controlling my character and while the games can influence me in certain ways, in the end I choose how I would like the game to go [..] I do my best to project my own choices onto my in-game character for full immersion.” (Bella)

For a few of the participants, this autonomy also allowed them to make choices that they would not ordinarily make in everyday life, such as stealing a car or goading an enemy into a fight. This seemed to be experienced as cathartic as well as fun, which would represent the fulfilment of autonomy as a basic psychological need (77).

“It gives me the option to make reckless choices and not worry so much about the consequences given they don't [affect] my real life.” (Ryan)

##### 3.1.1.2 Social connectedness

One of the most keenly felt restrictions imposed in response to COVID-19 was social distancing, which involved maintaining physical distance from anyone outside one's household. The extent of social distancing ranged between countries and in levels of severity, but resulted in feelings of isolation (78, 79), which represents the frustration of relatedness needs according to SDT (80). Participants described how video games combat loneliness and disconnection by providing a remote way of actively socialising with others.

Some participants already used video gaming prior to the pandemic to connect with friends in different countries. The fact that the pandemic resulted in more people engaging in socialising through video games was seen as positive.

“I had already used online gaming as a social outlet due to living far from friends in other states, so the increase in usage across the country (and world) has helped me socialize with others even more.” (Bella)

Other participants, who had less experience of gaming prior to the pandemic, were pleasantly surprised by online gaming communities and how they felt a sense of connection from strangers engaging with their content, representing a greater fulfilment of relatedness needs despite the frustration of physical relatedness needs.

“I'm also a member of a Facebook group page for Animal Crossing and I find it really interesting to see other people's islands – I actually posted a picture of my character in a graduation gown on my island when my graduation should have been as it was cancelled because of the pandemic. Thousands of people liked this picture and congratulated me, which was really nice. There's a real sense of community!” (Paige)

Participants emphasised how these interactions were facilitated through video streaming platforms like YouTube and Twitch, which “*can create a community surrounding a shared love of video games which has definitively helped me get through the isolation of this quarantine*” (Jackson). This was contrasted against other forms of social media, which were understood to be “*a toxic environment where you can compare yourself to others. Online gaming is generally a more friendly atmosphere*” (Will).

Even within households, video games provided participants with opportunities to deepen their relationships with their families and partners through the shared experience of their chosen games. Participants reflected that this was not necessarily something they would have done outside of the lockdowns.

“With the lockdown and us all being at home, my children were already into Minecraft and I had resisted playing until then [..] when lock down hit I just started playing it with them and entering their world a bit more.” (Michael)

“My wife and I played this together as a co-op game and it was a really good alternative to the other things we did together pre-lockdown, such as going out for a meal or to the cinema. Again, I felt like I was sharing something with her through the game.” (Stephen)

Participants also felt this sense of social connection towards characters in video games; for some, this meant that character development and relationship-building were key factors that drove their engagement with games.

“With Animal Crossing New Horizons, I love the characters that are my residents on my island. It really feels like a little community.” (Mia)

##### 3.1.1.3 Progression and development

Lockdowns and resultant restrictions led to widespread disruptions to everyday life, including attending school and working. With many individuals 'stuck' inside and facing uncertainties regarding their future, this led to feelings of stagnation and a lack of development opportunities (81). In contrast, participants described how video games provided them with a sense of purpose and progress, allowing them to continue developing and refining their skills in ways that provided a sense of achievement and growth. This was experienced through levelling up characters, completing quests, and winning more games. The ability to succeed through the game would represent the fulfilment of competence needs (61), which many individuals felt they were unable to do during the COVID-19 lockdowns.

“The sense of accomplishment and progression. I know I am getting better at the game as I understand the mechanics and systems of the game, and the game visually tells me that I am getting better. There is a tangible thing that I can see that reflects my improvement. Whether that be I reach higher levels in an RPG or get better rankings and win more matches in a Fighting Game.” (Jackson)

“Your account gains levels but you also gain Mastery for every champion. I recently got to a new level with my favourite champion and it felt amazing as a sense of achievement but also gave me the drive to play the character more.” (Mia)

For some, these in-game accomplishments translated into real feelings of progression and skill development, providing additional confidence and motivation for real-life tasks and challenges.

“Accomplishing mini-goals in video games gives me a huge boost in confidence and ability to know I can achieve goals with practice and work.” (Bella)

“With the games that I play most, the content that is played in a group can often be chaotic and requires quick thinking or adjustment in order to be successful. With this I believe it has made me more capable of staying focused on the task and calm in stressful scenarios, or managing other people while in such situation.” (Chloe)

#### 3.1.2 Theme 2: proactive and protective: video games as a coping mechanism

Participants reflected on how they used video games as a coping mechanism throughout the pandemic, both to protect and enhance their mental health and well-being. Three subthemes were generated: (2.1) Gaming to escape the pandemic, (2.2) Gaming to unwind, and (2.3) Gaming to support mental well-being.

##### 3.1.2.1 Gaming to escape the pandemic

For many participants, video games provided them with the opportunity to escape from “*the mayhem of reality*” (Paige) and be transported to an entirely different world. This was seen as particularly vital during the context of the pandemic, where significant anxiety, stress, and uncertainty was experienced on a daily basis.

“I can “zone out” of the real world and fully immerse myself in a virtual world and prioritise that for a few hours.” (Emily)

“I honestly believe that video gaming is more important than ever. It allows for people to distract themselves from all of the stuff that['s] going on both in the world and in their nation [..] in engaging with the virtual worlds and stories that are being told through video games we are in a much better mental state than being dragged down by all of the negativity in the current media waves.” (Jackson)

However, participants reflected that some games were better placed to afford this escapism than others, with fantasy RPGs and historical action-adventure games preferred over genres such as horror (e.g., The Last of Us). The ability to be immersed in a story far removed from reality was a key component of this protective mechanism.

“As for fantasy content, I prefer to interact in a game environment that is as far away from “real life” as possible due to immersion in a whole new world I only get to experience online.” (Bella)

“RDR2 [Red Dead Redemption 2]: A total distraction from life's problems given the scale and subject matter of the game, something totally removed from modern life.” (Ryan)

“Nothing too grim (e.g., The Last of Us) either, since I want some escapism from games.” (Stephen)

##### 3.1.2.2 Gaming to unwind

Alongside escapism, participants described the importance of video games in allowing them to de-stress. The uncertainty experienced during the pandemic was a significant proponent of this stress, alongside changes to everyday activities, like increased childcare and remote working. Video games were seen as an easy and enjoyable way to relax, with some participants specifically purchasing consoles at the outset of the pandemic for this reason.

“They can be an easily accessible way of unwinding and switching off from whatever it is that I need to switch off from. My [job] does not involve particularly pleasant subject matter and so it can be helpful to switch on a game and redirect my thoughts.” (Michael)

“I think at first video games curbed my anxiety and de-stressed me. I actually purchased a Nintendo Switch during the pandemic just so I could play Animal Crossing and my partner commented that I seemed less stressed out.” (Paige)

Games that were passive and required less effort were particularly appreciated as a way to de-stress, potentially because they required less mental energy to engage in. Participants expressed how tired they had become due to the pandemic and appreciated opportunities where they did not have to focus.

“Lockdown has been really tiring [..] my work has got more difficult because it's hard to focus, but I feel under the same pressure as before [..] it's been a tough time.” (Adam)

“The simplistic and repetitive nature of this game combined with its freedom to be creative allows for the more mellow and relaxing moments of playtime during this quarantine.” (Jackson)

##### 3.1.2.3 Gaming to support mental well-being

Participants also used video games more actively to support their mental health and well-being. In the context of anxiety disorders, video games were seen as being able to provide a safe, controlled environment where participants could build their confidence and practice exposure at their own pace.

“Video games really help my social anxiety when trying to meet new people, and provide me with safe areas to explore myself when interacting online with others, which has been the biggest benefit overall.” (Bella)

Video games were also used as a way to cope with stress, with some participants using the promise of later gameplay as a reward and motivator to complete tasks.

“If I'm having a stressful day, say at work for example, I always think something along the lines of “get through today then you can play games when you get home”. This gives me something to look forward to.” (Mia)

#### 3.1.3 Theme 3: to game or not to game? conflicts faced by lockdown gamers

This final theme summarises some of the conflicts expressed by participants regarding video gaming; whilst the positive aspects of the hobby dominated many of the interviews, there was explicit and implicit discussion of some of the negatives experienced. This is captured through three subthemes: (3.1) An enjoyable hobby or a “good enough” alternative?; (3.2) Guilt-free or guilt-ridden?; and (3.3) Helpful or harmful?

##### 3.1.3.1 An enjoyable hobby or a “good enough” alternative?

Many participants expressed how they enjoyed playing video games as a hobby, particularly in the context of lockdown, as it enabled them to engage in activities they may have otherwise been unable to do.

“It was kind of like going on an adventure with [the children] without leaving the house.” (Michael)

“I played online for the first time so I could try Streets of Rage 4 (co-op) with my friend in [country]. He had to cancel his scheduled visit to come see us over Easter, so it was really nice to at least play through the game with him. We talked on Zoom at the same time.” (Stephen)

That said, some participants seemed to position video games as a “good alternative” to in-person activities and not necessarily what they would prefer to do if given the option of both. In this way, video gaming seemed less like an enjoyable hobby and more like an alternative which was “good enough” but not what would have been chosen under ordinary circumstances.

“I prefer getting out and visiting historical places and actually experience some of the type of places I see in the games (such as battlefields, historical houses, playing football, etc.).” (Ryan)

“I was near the end of my assessments for the year at university and other summer plans were unable to go ahead, so I could play more games than usual due to more free time.” (Chloe)

“It became one of the main things to do every evening in our household, since other options were either unavailable or weren’t safe.” (Stephen)

##### 3.1.3.2 Guilt-free or guilt-ridden?

For some participants, the COVID-19 lockdown provided them with the opportunity to play video games to a greater extent than they would normally be able to. This was due to increased free time due to circumstantial changes like furlough and working from home, removing the need to commute.

“I was made redundant and was quarantined throughout lockdown for 3 months and this was my “hobby”, so ended playing many more hours than usual. Also felt like “there will not be another opportunity where this would happen again” and I enjoy spending time online gaming so enjoyed spending more time with friends and progression.” (Will)

This change was eagerly received by some, allowing them to participate in “*guilt-free*” (Stephen) gaming and return to more labour-intensive games that they did not typically have time for in their everyday life.

“I've been playing League of Legends on and off for a few years so it's a game I usually come back to every once in a while. Quarantine was a good time to come back to the game since playing the game can take a lot of time up in the day.” (Mia)

However, throughout the interviews, there was an underlying narrative of worry regarding increased gaming. Participants described internal feelings of guilt provoked by deciding to play video games instead of other activities, which were understood to be more productive and worthwhile. Some participants even went as far as to suggest that they were “*wasting my life*” (Paige) when video gaming. In this way, there seemed to be an internal battle between enjoying the opportunity to game more whilst also feeling guilty about doing so.

“On the rare occasions where I have time to myself, I'll often play games but constantly feel like I should be exercising or doing something productive instead.” (Ryan)

##### 3.1.3.3 Helpful or harmful?

Whilst the many benefits of video gaming were discussed at length, such as increased autonomy, the opportunity to socialise, and improved mental well-being, participants also acknowledged that video games could be harmful in certain situations. In particular, participants emphasised the addictive element of gaming, which could distract from real-world tasks and responsibilities and have a felt detrimental impact.

“I think at first I became quite obsessive with gaming at the beginning of the pandemic so it perhaps distracted me from work.” (Paige)

While video gaming during lockdown largely seemed to reduce stress, participants reflected that in certain situations, gaming could increase levels of stress.

“If I'm already quite wound up, something on a video game going wrong can just tip me over the edge.” (Ryan)

“Sometimes I can find the games increase my stress when there is a particularly tense situation I am playing through.” (Chloe)

The same could also be said for mental health; while gaming largely seemed to provide benefits for participants, in certain situations, it could be detrimental to mental well-being. This was identified as a particular issue in the context of toxicity from other players and less due to the games themselves.

“The only time video games really affect me negatively is when I'm playing League of Legends and people are being toxic towards me. I've got better at dealing with it but it still affects me.” (Mia)

## 4 Discussion

Analysing the experiences of video gamers during the COVID-19 lockdown illustrates the far-reaching implications of using video games as a stress-relief mechanism, which makes the lockdown experience an effective “case study” for times of social difficulty or crises. While the use of COVID-19 provides important teachable moments of the need to maintain and develop social relationships in digital environments, the current study further exemplifies the detrimental impact that social isolation can have on psychological health and the role that video gaming can play as a preventative measure. The lessons learned in the lockdown are also applicable to individuals experiencing limited social contact (82, 83) or individuals who consistently experience the frustration of basic psychological needs (84).

The current study provides an intriguing window into the worlds of video gamers during the pandemic, generating insights into the benefits and pitfalls of using video games to help navigate the psychological stressors of COVID-19, particularly those brought about by social distancing measures. The study's first theme (Providing what lockdown took away) may be considered through the lens of self-determination theory (39). Self-determination theory argues that individual psychological health and well-being rely on meeting three basic psychological needs: autonomy, competence, and relatedness. During a time of greater uncertainty with restrictions on individual liberty, these psychological needs would be more difficult to satisfy. Consequently, participants discussed how playing video games gave them a sense of control and agency restoration, a feeling of community and connectedness with others, and a sense of purpose and progress. Playing video games provided participants with an alternative way to satisfy these important psychological needs and maintain positive well-being during an especially challenging time. These findings could thus be interpreted as a compensatory response to the stressors of COVID-19 (85).

Theme 2 (Proactive and protective: Video games as a coping mechanism) suggests video games were used as both reactive and proactive coping strategies during the pandemic. A commonly recounted reactive strategy was one of escape avoidance (17), whereas a more proactive strategy included future-oriented coping, for example, the promise of game time as a reward for the anticipation of a difficult day. While avoidant strategies may help reduce short-term stress, they are considered less effective in the long term and even as a form of maladaptive coping [e.g., (86)]. Moreover, certain groups (e.g., women, younger adults, and people of lower socio-economic status) were found to be more reliant on avoidant coping during the pandemic (87). Given video games' immersive and time-distorting properties (88), there may be a danger of becoming too reliant on them as a coping mechanism, particularly if this entails distancing oneself too much from the stressor. One would also expect this to be the case if individuals were using video games as an avoidant coping strategy during other forms of stress-inducing events, such as putting off completing a difficult assignment at university.

Theme 3 (To game or not to game? Conflicts faced by lockdown gamers) speaks to the tension between viewing video games as a worthwhile leisure activity with psychological, social, and developmental benefits vs. a time-wasting activity which detracts from more “important” pursuits. In the past, video gaming has often received negative press, particularly regarding its potential for harm, for example, its alleged impact on aggression (40). It is difficult to know, particularly for older gamers, the extent to which this broader societal narrative around video game harms has been internalized, and this may represent a fertile avenue for further investigation.

A further consideration is that the COVID-19 experience affected everyone in different ways, such as those who identify as a gender or sexual minority (89), which likely increased the salience of stress during this time. Video gaming was a popular activity for those most in need of a coping strategy (90) to deal with the stress associated with life during the COVID-19 pandemic (87) due to gaming's ability to help relieve stress (68). This compensatory use of video gaming would have unintentionally created a risk of increased “addictive” play behaviours and excessive play to relieve the stress of the pandemic (91, 92). While it has been recorded that gaming was overused due to factors such as social isolation (93), it can be argued that social gaming can be used to ameliorate this (94). Therefore, it is crucial that gamers should avoid the *overuse* of gaming as a compensatory stress-management strategy, being mindful of how video gaming can be used in moderation alongside other activities and making use of digital health services to report concerns in play behaviours.

Considering that the World Health Organization considers the next global pandemic to be a “matter of when, not if” (95), the efforts of this study to analyze mental health and video game behaviours during a global pandemic may provide useful insights into how individuals can use video gaming to regulate mood and stress when under extreme psychological pressure. Indeed, it illustrates the capacity of video gaming, when used healthily, to fulfil basic psychological needs and provide a means of coping with heightened levels of stress external to the video game, which is likely to be a key lesson for future health epidemics and beyond.

However, this study is not without its limitations. First and foremost, the data gathered was influenced by the lack of physical social interactions. While maintaining COVID-19-based health advice was of the utmost importance, this limited the data collection process to online-only methods. In addition, the nature of qualitative research means that researcher subjectivity is an inherent part of the research process. However, this is not a limitation in itself but is instead viewed as “a resource for research, rather than a threat to be contained”, with the view of “meaning and knowledge as contextually situated, partial and provisional” (96, pg. 2). As such, the findings should be considered within the context of researcher subjectivity, but this does not diminish the rigour of the study. Furthermore, the recruitment of a largely young male sample, as noted previously, may constrain the transferability of the findings and omit perspectives from individuals of other genders, age groups, and cultural backgrounds, which we encourage for future research endeavours.

We acknowledge that gaming experiences are often defined by the intersection between, and social construction of, race, gender, and culture (97, 98), including the social construction of the gamer identity or the perceived taboo surrounding “female gamers” (99). This was not explicitly reflected in the participants of this study, which captured a more traditional male understanding of the video gaming experience. While quantitative efforts demonstrated a relatively universal experience on an international scale (100) and with adolescents (90), the results of this study should be interpreted with a need for greater consideration of intersectionality in mind. In particular, we recommend greater efforts to represent voices and perspectives in gaming that have previously faced exclusion, such as voices of colour or LGBTQIA+ identities (97).

## 5 Conclusion

To conclude, video gaming during the pandemic served as an important coping mechanism for some individuals, providing them with opportunities for social interaction, both within their household and outside of it, a world in which they could become immersed and live autonomously, and a sense that they had progressed, achieved, and grown. While this undoubtedly carried risks of developing unhealthy gameplay behaviours, such as excessive play patterns, video gaming offered a digital compensatory stress-management strategy.

Based on the findings of this qualitative study and the documented experience of the COVID-19 pandemic at large, we argue that there are teachable moments for digital mental health practitioners. For example, we recommend greater cooperation between video game developers and digital mental health practitioners, developing greater awareness of “unhealthier” methods of play, such as excessive play, or recognizing “addictive” play at a national level. Alternatively, there could be greater involvement by the video game industry to help steer video gamers towards games that help satisfy basic psychological needs (games rich in opportunities to satisfy needs for autonomy, competence, and relatedness, such as role-playing games or games with multiplayer capabilities), which would benefit those in danger of social isolation and greater stress. It would also be sensible to include the benefits and drawbacks of video gaming in crisis intervention frameworks, offering an accessible stress-management strategy if used in moderation, that is applicable in times of social upheaval. Ultimately, any effort towards identifying the use of games to manage stress must also include greater awareness of balanced uses of video gaming to avoid further harm to psychological health.

While video games are often maligned in the mainstream media, this study adds to the growing research base for their benefits and suggests that video gaming may be an important psychological buffer against the negative impact of heightened stressors, both in future global health events and for socially isolated or need-frustrated individuals.

## Data Availability

The raw data supporting the conclusions of this article will be made available by the authors, without undue reservation.
