# Supplementary material for: “It's a good distraction from the mayhem of reality”: a reflexive thematic analysis on the role of video games to support coping during a crisis
Source: Front Digit Health. 2025 Aug 18;7:1608322. doi: 10.3389/fdgth.2025.1608322 (PMC12400524; doi:10.3389/fdgth.2025.1608322)
Supplement: Supplementary file 1 [file Datasheet1.pdf]

Interview schedule

[Start Interview]

Hello, my name is George and together with some colleagues from several different universities, we're conducting some research about video game behaviour within the context of the COVID-19 lockdown. The coronavirus places us all in in a unique set of circumstances, which would naturally alter some behaviours (including video gaming patterns), and it is this change in behaviour that we wish to study. We're hoping that with this research, it helps people to understand how social contexts define and change particular aspects of human behaviour, specifically the role that video games play or have played in a "quarantine lifestyle". This interview should take approximately 30-60 minutes, do you consent to be a part of this research, and for me to ask you questions?

[consent gained]

Great, I'll begin by asking some basic questions about yourself:

1) What is your age?
2) What is your gender identity?
3) What is your current employment status, (including 'furloughed' status), and has this changed since the quarantine began?
4) Are you considered a 'key worker'?

Thanks for answering those. Moving on, I'm going to ask you some questions about your current video game playing:

1) How many hours do you spend playing video games per week? How many days per week do you normally play? What's the longest you play a game for a single session?
2) What would you say are your top three video game title during quarantine? Why do you like these games in particular?
3) Are there any particular video game genres that you prefer? Why is that?
4) Did you find yourself playing video games more, less, or the same during quarantine? Why do you think that is? How does that make you feel?

Ok, thanks for answering those. I'm now going to ask you some questions about video games in general:

1) What kinds of benefits would you say video games offer you, compared to other digital mediums such as social media?
2) Do you prefer video games over other leisure activities (physical or otherwise)? Why do you think that is?
3) What makes the video games that you play worth playing (i.e. why you keep playing; social interaction; competency, etc.)?

Ok, thanks for answering those. Now I'm going to ask you some questions about your experiences with video gaming during the COVID-19 pandemic:

1) Have you found the COVID-19 pandemic difficult to deal with? Please explain.
2) Do video games affect your levels of stress or anxiety? In a positive or negative way? Why do you think that is?

3) How socially connected do you feel playing a video game? How would you increase or decrease feelings of social connectivity in a video game?
4) Do you feel like video gaming gives you the ability to make choices freely? In what ways?
5) How does video gaming impact your ability to achieving a goal/s?
6) Do you feel like video gaming has impacted on your ability to cope with stressful situations? In what ways?
7) Is there anything else you would like to add/discuss?

Great, thanks for answering, this concludes the interview. Thank you very much for taking part in this research and taking the time to answer these questions. Is there anything else you would like me to know before we finish?

Once again, thanks for taking part and your information will be anonymous going forward.

[End Interview]
