# Supplementary material for: “It's a good distraction from the mayhem of reality”: a reflexive thematic analysis on the role of video games to support coping during a crisis
Source: Front Digit Health. 2025 Aug 18;7:1608322. doi: 10.3389/fdgth.2025.1608322 (PMC12400524; doi:10.3389/fdgth.2025.1608322)
Supplement: Supplementary file 2 [file Datasheet2.pdf]

Basic Questions

1) What is your age?

2) What is your gender identity?

3) What is your current employment status, (including 'furloughed' status), and has this changed since the quarantine began?

4) Are you considered a 'key worker'?

Current Video Game Play:

5) How many hours do you spend playing video games per week?

6) How many days per week do you normally play?

7) What's the longest you play a game for a single session?

8) What would you say are your top three video game title during quarantine?

9) Why do you like these games in particular?

10) Are there any particular video game genres that you prefer, and why?

11) Did you find yourself playing video games more, less, or the same during quarantine?

12) Why do you think that is?

13) How does the change (or lack of change) in video game playing time make you feel?

General Video Game Questions:

1) What kinds of benefits would you say video games offer you, compared to other digital mediums such as social media?

2) Do you prefer video games over other leisure activities (physical or otherwise)?

3) Why do you think that is?

4) What makes the video games that you play worth playing (i.e. why you keep playing)?

Video Gaming and COVID-19:

1) Have you found the COVID-19 pandemic difficult to deal with? Please explain.

2) Do video games affect your levels of stress or anxiety in a positive or negative way?

3) Why do you think that is?

4) How socially connected do you feel playing a video game?

5) How would you increase or decrease feelings of social connectivity in a video game?

6) Do you feel like video gaming gives you the ability to make choices freely, and in what ways?

7) How does video gaming impact your ability to achieving a goal/s?

8) Do you feel like video gaming has impacted on your ability to cope with stressful situations and in what ways, if any?

9) Is there anything else you would like to add/discuss?
